# It’s All About the CREY!

**DOI:** 10.1093/function/zqae021

**Published:** 2024-04-22

**Authors:** Stephanie Franzén

**Affiliations:** Department of Medicine, Division Nephrology, Section Cardio-Renal Physiology and Medicine, University of Alabama at Birmingham, Birmingham, AL 35233, USA; Department of Surgical Sciences, Division Anesthesiology and Intensive Care, Uppsala University, Uppsala, 75185, Sweden

How do you do it? How do you make time for everything? As a young, busy, and socially active scientist, peers, and students continuously ask me the same questions. And the systematic answer: It’s all about the CREY.

Circadian Rhythm EfficiencY (CREY) is a model I have defined as I go through life to juggle it all. This is a systematic, individual tool in order to be productive in the most efficient way but still make time for everything outside of being a scientist. Making time for recharging. Basically, it’s all about streamlining your 24 h. Bear with me, I'll explain it in detail.

The inner 24 h biological clock, also known as circadian rhythm, has a significant and essential impact on basic physiology and pathophysiology. Circadian rhythm is controlled centrally by the suprachiasmatic nucleus. A zeitgeber (factor regulating circadian rhythm) with direct impact on this central clock is sunlight, which inhibits melatonin production. As a person living at a very northern latitude, I can acknowledge that severe shifts in daylight have a major impact on physiology. This is more commonly known as seasonal affective disorder.^1^ Additionally, it’s been shown that patients in the intensive care unit (ICU) have significantly disrupted circadian rhythm as they are constantly disturbed during all 24 h.^2^ This most likely contributes to more profound organ dysfunction and prolonged stay in the ICU. Illness severity in the ICU was actually correlated with maximum light exposure during nocturnal hours.^3^ Other factors regulating the circadian rhythm are the peripheral clocks located in different organs. They can be regulated by the central clock but may also have independent rhythms. For example, blood pressure is normally higher during the day and lower during nocturnal hours, and people who do not have this oscillation in blood pressure are more prone to develop cardiovascular disease.^4^ A recent study interestingly showed that food intake could act as a zeitgeber for blood pressure oscillation.^5^ The kidneys also have a profound circadian rhythm.^6^ Studies have shown that renal injury and cardiovascular dysfunction are present when any of the core regulating genes for circadian rhythm are knocked out.^7^ Also, a study on mice showed that coronary ischemia reperfusion injury was more severe during the sleep-to-wake transition than during the wake-to-sleep transition.^8^ This phenomenon has stimulated a discussion on whether myocardial infarctions during the morning have a higher mortality rate than if they happen during the evening. This might also be the case with other ischemic injuries such as stroke. Taken together, there is abundant evidence that disrupting the circadian rhythm has detrimental effects on physiology but also that cells are more or less susceptible to injury at certain times.

Circadian rhythm is not only the focus in physiology but also an important factor in cognitive function. Intrinsically photosensitive retinal ganglion cells (ipRGC) have been suggested to be the main mediator in light-dependent cognitive function.^9^ Cognitive function includes thought, reason, decision-making, and memory, which all define a person’s behavior. A comprehensive review discussed how light affects cognitive function via ipRGC.^10^ This pathway indirectly affects oxytocin release, and also the cholinergic neurons in the forebrain, which then influences awareness and attention. Hence, it makes sense that seasonal affective disorder presents with depression, exhaustion, and a lack of productivity.^1^ However, the rhythm of cognitive function is likely individual since some people are night owls and some people are early birds.

How the cognitive rhythm oscillates is most likely individual. I have come to learn that my cognitive rhythm oscillates on a 6 h cycle. Hence, I have two periods of enhanced cognitive function during the diurnal hours. I call it cognitive productive rhythm (CPR) and when individualized, it can be used to streamline your 24 h. Take a moment and think about it. When during your diurnal hours are you most productive and efficient? And the opposite is equally important, during which hours is your brain a fog and any work done during these hours is just poorly executed? Once these periods of efficient work and non-efficient work are defined, ergo your CPR, a CREY cycle can be created.

Let me be more specific and give my CPR and CREY as an example (Figure 1). I have realized that I am extremely productive with work between 8 am and 1 pm and between 8 pm and 11 pm. During the hours in-between, my brain simply does not function in a cognitive efficient-wise manner. So, based on this knowledge, I allow myself to work during my high-efficiency hours and to recharge during my low-efficiency hours. For example, during the low-cognitive hours I exercise, meet friends, run errands, or just do whatever I feel like that particular day. In this way, creativity and productivity return 6 h later. Importantly, this is according to *my* preferences and take note: Everyone has a different CPR. Hence, everyone’s CREY is different. Maybe you have two different CREY depending on whether it is an experiment-day or a computer-day. Do not waste the low-efficiency diurnal hours; use them to recharge!

**Figure 1.**
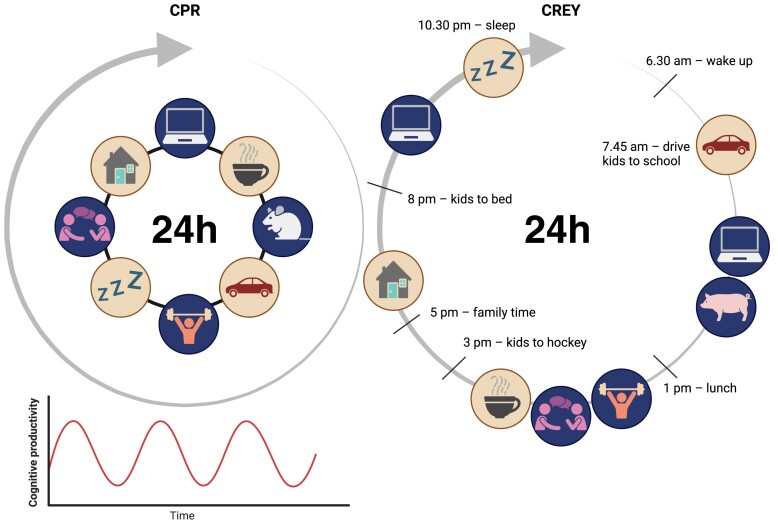
Schematic overview of my circadian productive rhythm (CPR, left) that oscillates on a 6h cycle, and a examples of what one need, want, and have to do each day. This translates into my specific circadian efficiency rhythm (CREY, right). This figure was created with biorender.com.

The general take home message here is that not only physiology but cognitive function oscillates. Streamlining the 24 h and still making time for everything is attainable in just a few steps. First, define your CPR—when during the diurnal hours are you most productive? Realize your individual cognitive oscillation. The second step is to design your individual CREY so that the 24 h includes everything you need, want, and have to do. The final and most difficult step is to accept it. By pushing yourself to work or do important things when cognitive function is low, you are likely disrupting the cognitive oscillation. The hypothesis is that by maintaining the CPR, high-efficiency hours will become even more productive as the brain is allowed to recharge in-between.

So, how do we make time for everything? It’s all about the CREY.

## Data Availability

No data was generated with this editorial.
